# Protective Effects of* Dracocephalum heterophyllum* in ConA-Induced Acute Hepatitis

**DOI:** 10.1155/2016/2684321

**Published:** 2016-07-25

**Authors:** Wei Zheng, Qilan Wang, Xiaohua Lu, Qiangqiang Shi, Junhui Zou, Yanduo Tao, Pu Wang

**Affiliations:** ^1^Shenzhen Institutes of Advanced Technology, Chinese Academy of Sciences, Shenzhen 518055, China; ^2^Institute of Military Veterinary Medicine, Academy of Military Medical Science, Changchun 130122, China; ^3^Northwest Plateau Institute of Biology, Chinese Academy of Sciences, Xining 810001, China

## Abstract

*Dracocephalum heterophyllum* (DH) is a Chinese herbal medicine used in treating hepatitis. However, the protective effects and pharmacological mechanisms of DH in hepatitis are unknown. In this study, we found that pretreatment with DH extract significantly ameliorated liver injury and suppressed the production of inflammatory cytokines, including tumor necrosis factor (TNF-*α*) and interferon-*γ* (IFN-*γ*) in Concanavalin A- (ConA-) induced hepatitis (CIH). DH recruited more CD11b^+^ Gr1^+^ myeloid-derived suppressor cells (MDSCs) to the liver and suppressed infiltration of macrophages (Kupffer cells) in the liver. The present work explores DH as an effective hepatoprotective medicine to inhibit inflammation and liver injury caused by hepatitis.

## 1. Introduction

Hepatitis, a global health problem, is caused by viral infections, autoimmune diseases, fatty liver diseases, and metabolic disorders. Acute hepatitis is characterized by strong inflammation, which induces hepatocyte death and can lead to liver failure [1, 2]. Concanavalin A- (ConA-) induced hepatitis is an appropriate animal model for drug research and immune-mediated liver injury for human hepatitis. Induction of hepatitis by ConA requires interaction between T cells and Kupffer cells; both T cells and Kupffer cells are responsible for liver damage in any type of acute hepatitis [3]. Kupffer cells play an important role in T cell activation-induced liver injury by contributing to tumor necrosis factor (TNF-*α*) production. Excessive activation of macrophages induces liver damage and is the most common cause of mortality during HBV and HCV infection [4, 5]. The depletion of Kupffer cells attenuates the production of hepatic TNF-*α* and interferon-*γ* (IFN-*γ*), lessens the infiltration of inflammatory cells in the liver, and protects mice from ConA-induced liver injury [6–11].

Myeloid suppressor cells (MDSCs) represent promising therapeutic targets in the treatment of liver diseases, with the liver being an important site for MDSCs accumulation and differentiation under various liver conditions. In acute hepatitis, MDSCs protect against liver injury by suppressing the activation of T cells and Kupffer cells and increasing the number of Treg cells. It is therefore helpful to increase hepatic MDSCs numbers for the treatment of patients with acute hepatitis [2, 4].

Currently, effective drug treatment options for hepatitis are limited. DH has been a traditional Chinese herbal medicine for hepatitis, but the effects of DH protection against liver damage remain unclear. In this study, we performed experiments to determine the therapeutic effects of DH on liver inflammation and injury, and we investigated the cellular and molecular changes in CIH treated by the administration of DH extract.

## 2. Materials and Methods 

### 2.1. Mice and Ethics Statement

Female BALB/c mice (6–8 weeks old) were obtained from Vital River Lab Animal Technology (Beijing, China). The mice were housed in an animal facility at a humidity of 40–60% and a temperature of 22–24°C with a 12 h alternating light and dark cycle. Five mice were housed in a standard polypropylene cage with stainless steel top grill having facilities for SPF food (approval IDs: SCXK 2014-0010) and drinking water bottle. The mice were observed once a day. These measures guaranteed that the mice were not expected to die.

All studies were performed according to the National Institutes of Health Guidelines and were also approved by the Animal Care and Use Committee of Shenzhen Institutes of Advanced Technology, Chinese Academy of Sciences (Approval IDs: SCXY 2012-0119) and used in accordance with regulations and guidelines of this committee. All efforts were made to minimize suffering and distress. At the end of the experiment, mice were euthanized by cervical dislocation method.

### 2.2. Preparation of DH Extract

The medicinal DH was powdered with a mechanical grinder. The powdered DH was macerated in 95% ethanol and allowed to shake for 2 h and then was filtered through a filter paper. The maceration process was repeated 3 times. The filtered extract was concentrated in a rotary evaporator at 40°C and then freeze drier was used to remove ethanol and water. The dry extract was stored at 4°C until used. DH extract was dissolved in dimethyl sulfoxide (DMSO) (AMRESCO, USA) and further diluted with Phosphate Buffered Saline (PBS). The working concentration of extract is 2.5 mg/mL. The final concentration of DMSO in injection solution was <0.1% (v/v).

### 2.3. Treatments of Mice with DH and ConA

Six- to eight-week-old female BALB/c mice were randomly divided into four groups with each group containing 5 mice. Control group mice were injected via the tail vein (*i.v.*) with PBS; DH control group mice were injected by intraperitoneal injection (*i.p*). With DH extract, ConA-treated group mice were injected* i.v.* with either a lethal dose (30 mg/kg) or a sublethal dose (15 mg/kg) of ConA (Sigma-Aldrich, USA), and DH-pretreated mice were injected* i.p.* with DH (20 mg/kg) 2 h before injecting* i.v.* with ConA.

In the survival rate study, mice were monitored every 4 h after injection of the lethal dose of ConA and euthanized at 24 h after injection. In the sublethal dose injection groups, blood was obtained through orbital plexus bleeding from each group at 8, 16, and 24 h after ConA administration. Serum alanine aminotransferase (ALT) and aspartate aminotransferase (AST) levels were measured by a transaminase kit (Sigma-Aldrich, USA) according to the manufacturer's instructions.

### 2.4. Histopathological Study and TUNEL

Sixteen hours after ConA injection, the mice developed significant liver injury [12]. Therefore, we choose this time span to examine liver pathology. The mice were euthanized by cervical dislocation method, and then liver tissues were fixed in 10% buffered formalin and embedded in paraffin. Tissue sections were cut and stained with hematoxylin and eosin (H&E) or TUNEL (terminal deoxynucleotidyl transferase- (TdT-) mediated dUTP-biotin nick end labeling) kit (Promega, USA) to observe the level of inflammation and tissue damage by light microscopy or fluorescence microscope.

### 2.5. Analysis of Serum Cytokines

DH-pretreated mice were injected* i.p. *with DH (20 mg/kg) 2 h before injecting* i.v.* with ConA (15 mg/kg). As has been reported previously, cytokines reached concentrations at different time points [13]. Blood was obtained through orbital plexus bleeding from each group at 8, 16, and 24 h after ConA administration. Serum concentrations of IFN-*γ* and TNF-*α* were determined using a specific enzyme-linked immunosorbent assay (ELISA) kit (Dakewe, China) according to the manufacturer's instructions.

### 2.6. Isolation of Liver Mononuclear Cells

Liver mononuclear cells (MNCs) were isolated as previously described. Livers were passed through a 200-gauge stainless steel mesh. The cells were suspended in 40% Percoll (Sigma-Aldrich, USA), then gently overlaid on 70% Percoll, and centrifuged at 1000 ×g for 30 minutes at room temperature. Liver MNCs were collected from the interphase of Percoll.

### 2.7. Flow Cytometric Analysis

Kupffer cells were stained with BV510-anti-CD45 and APC-anti-F4/80, and MDSCs were stained with PE-anti-CD11b and FITC-anti-Gr1 monoclonal antibodies (Biolegend, USA) for surface antigens according to standard protocol. The stained cells were analyzed using a flow cytometer (FACSCalibur; Becton Dickinson, Franklin Lakes, NJ), and the data was analyzed by FlowJo software.

### 2.8. Statistical Analysis

The results were analyzed by Student's* t*-test or analysis of variance. All data were shown as mean ± standard error of the mean (SEM). *P* value <0.05 was considered to be statistically significant.

## 3. Results

### 3.1. DH Protects against ConA-Induced Acute Hepatitis

**Figure 1:**
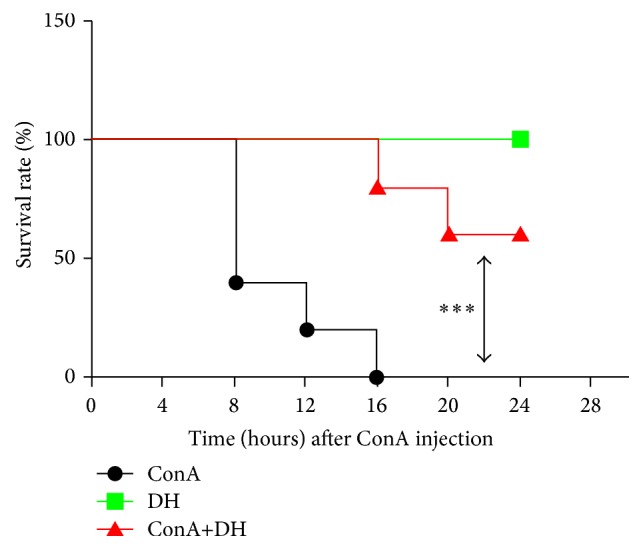
DH protects against ConA-induced acute hepatitis. Notes: female BALB/c mice (*n* = 10) were injected* i.p*. with DH (20 mg/kg body weight) at 2 h before the injection of a lethal dose of ConA (30 mg/kg)* i.v*. The survival rate was monitored at different times after ConA administration, with the data expressed as the mean ± SD (*n* = 10; ^*∗∗∗*^ *P* < 0.001 for ConA versus ConA+DH). ConA: Concanavalin A; DH:* Dracocephalum heterophyllum*.

**Figure 2:**
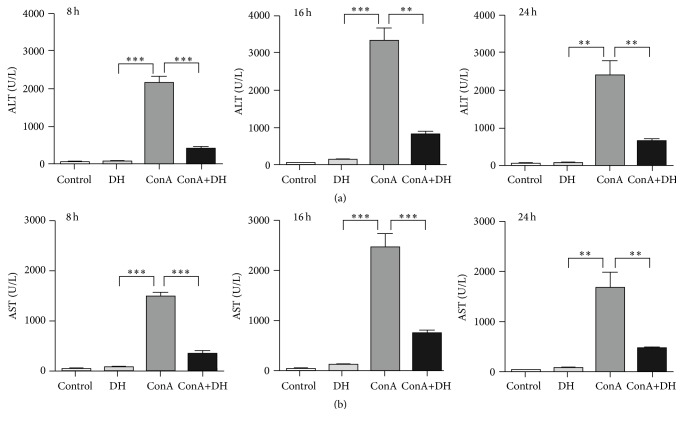
DH suppresses transaminase activity in ConA-induced hepatitis. Notes: female BALB/c mice were induced by intraperitoneal injection with DH (20 mg/kg of body weight) at 2 h before the injection of a sublethal dose of Concanavalin A (15 mg/kg of body weight) via the tail vein. Serum transaminase ALT (a) and AST (b) levels were determined 8, 16, and 24 hours after Concanavalin A injection. Data is expressed as mean ± SD (*n* = 3; ^*∗∗*^ *P* < 0.05 and ^*∗∗∗*^ *P* < 0.001). ALT: alanine transaminase; AST: aspartate transaminase.

**Figure 3:**
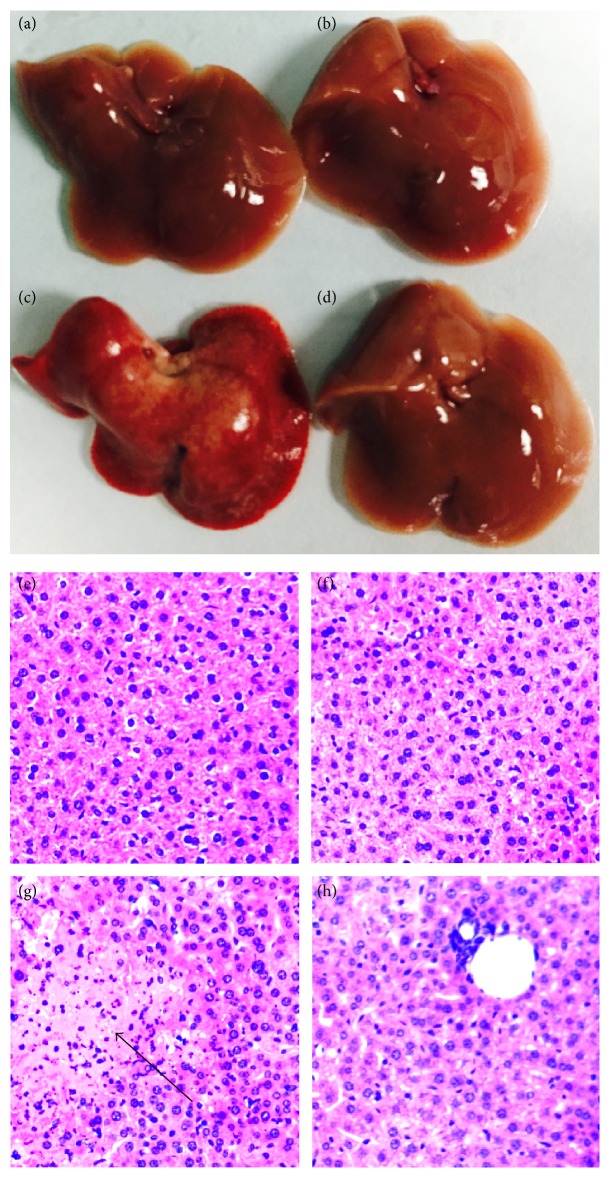
DH inhibits ConA-induced hepatitis. Notes: mice were injected* i.p.* with DH (20 mg/kg of body weight) at 2 h before the challenge of ConA (15 mg/kg of body weight). Mice were sacrificed at 24 hours after the ConA injection. The livers were harvested from Control (a), DH (b), ConA (c), and ConA+DH (d) injection mice, respectively. Liver tissues from Control (e), DH (f), ConA (g), and ConA+DH (h) groups were fixed and stained with hematoxylin and eosin (H&E). The arrow indicates massive cell death in the liver section. Original magnification ×200.* i.p.*: intraperitoneal injection; ConA: Concanavalin A; DH:* Dracocephalum heterophyllum*; H&E, hematoxylin and eosin.

**Figure 4:**
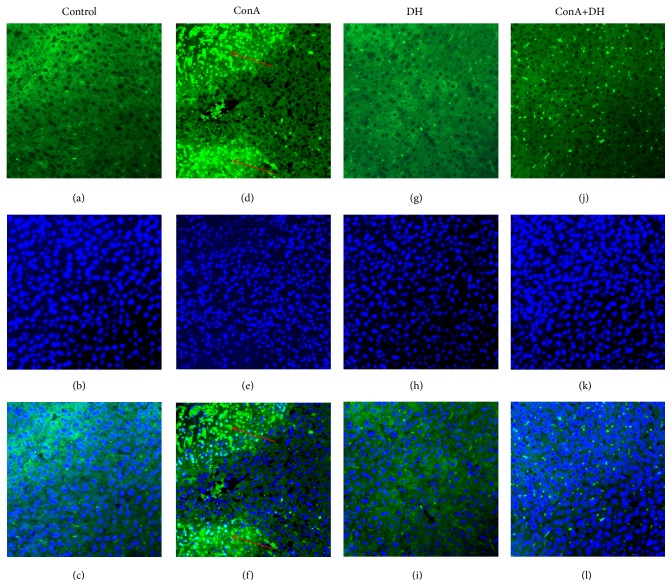
DH inhibits ConA-induced hepatic apoptosis. Notes: mice were sacrificed at 24 hours after ConA injection. The livers were harvested from Control, ConA, DH, and ConA+DH groups, respectively. Images showing TUNEL-labeled (green) apoptotic cells counterstained with DAPI (blue) in liver tissue sections. DAPI-stained sections (b, e, h, and k) label the nuclei, while TUNEL-labeling (a, d, g, and j) reveals apoptotic cells. Colocalization of TUNEL/ DAPI (c, f, i, and l) in liver sections. The arrows indicate an aggregate of apoptotic cells in liver section (d and f) of ConA injection group. Original magnification ×200. ConA: Concanavalin A; DH:* Dracocephalum heterophyllum*; TUNEL, terminal deoxynucleotidyl transferase- (TdT-) mediated dUTP-biotin nick end labeling; DAPI: 4′,6-diamidino-2-phenylindole.

To examine the therapeutic role of DH in hepatitis, we primarily investigated the effects of DH on mortality from acute hepatitis induced by ConA. Mice were pretreated with DH and then challenged with a lethal dose of ConA. Pretreatment of DH significantly increased the survival of mice that were challenged with a lethal dose of ConA when compared with untreated control mice (Figure 1 shows survival rate). The kinetics of hepatic damage was further determined in mice that were injected* i.v. *with a sublethal dose of ConA. Liver injury was evaluated by blood transaminase assays. As shown in (Figure 2), serum ALT and AST activities were significantly lower in DH-pretreated group than in nonpretreated group at 8, 16, and 24 h after ConA administration. As important evidence, anatomical and histological examinations were used to evaluated liver injury level (Figures 3(a)–3(d) show mice liver photos of different experimental groups, and Figures 3(e)–3(h) show hematoxylin-eosin staining). ConA-induced hepatitis was characterized by inflammatory infiltrates and widespread hepatocyte death. We observed more inflammatory infiltrates and widespread hepatocyte death in nonpretreated group. In contrast, minor hepatic damage and inflammatory infiltrates were observed in DH-pretreated group and the number of apoptotic cells was reduced in this group compared with nonpretreated group (Figure 4 shows TUNEL staining). These findings indicate that DH significantly reduces liver injury and release of ALT and AST in CIH. Altogether, the results show that DH effectively protects against liver injury.

### 3.2. DH Inhibits Production of IFN-*γ* and TNF-*α* in ConA-Induced Hepatitis

**Figure 5:**
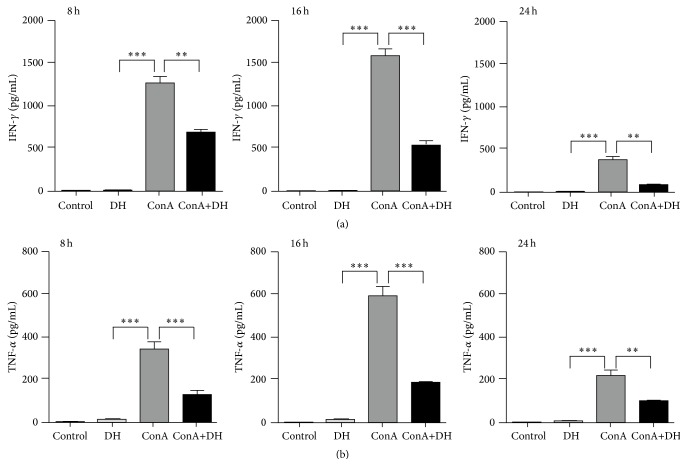
DH inhibits expression of IFN-*γ* and TNF-*α* in ConA-induced hepatitis. Notes: blood samples were collected from Control, DH-treated, ConA, and ConA+DH groups at 8, 16, and 24 hours after ConA injection. Serum levels of IFN-*γ* (a) and TNF-*α* (b) were detected by ELISA. Data is expressed as mean ± SD (*n* = 5; ^*∗∗*^ *P* < 0.01 and ^*∗∗∗*^ *P* < 0.001). ConA: Concanavalin A; DH:* Dracocephalum heterophyllum*; IFN-*γ*: interferon-*γ*; TNF-*α*: tumor necrosis factor; ELISA: enzyme-linked immunosorbent assay.

IFN-*γ* and TNF-*α* play critical roles in acute hepatic injury [14]. To explore the mechanisms of DH protection against liver injury, the effects of DH in IFN-*γ* and TNF-*α* production in ConA-induced hepatitis were studied. We measured serum level of IFN-*γ* and TNF-*α* by ELISA. The result showed that DH inhibited IFN-*γ* (Figure 5(a)) and TNF-*α* (Figure 5(b)) levels in serum in ConA-challenged mice. Thus, these results indicate that DH can suppress the expression of IFN-*γ* and TNF-*α* in CIH.

### 3.3. DH Recruits More MDSCs to Liver against ConA-Induced Hepatitis

**Figure 6:**
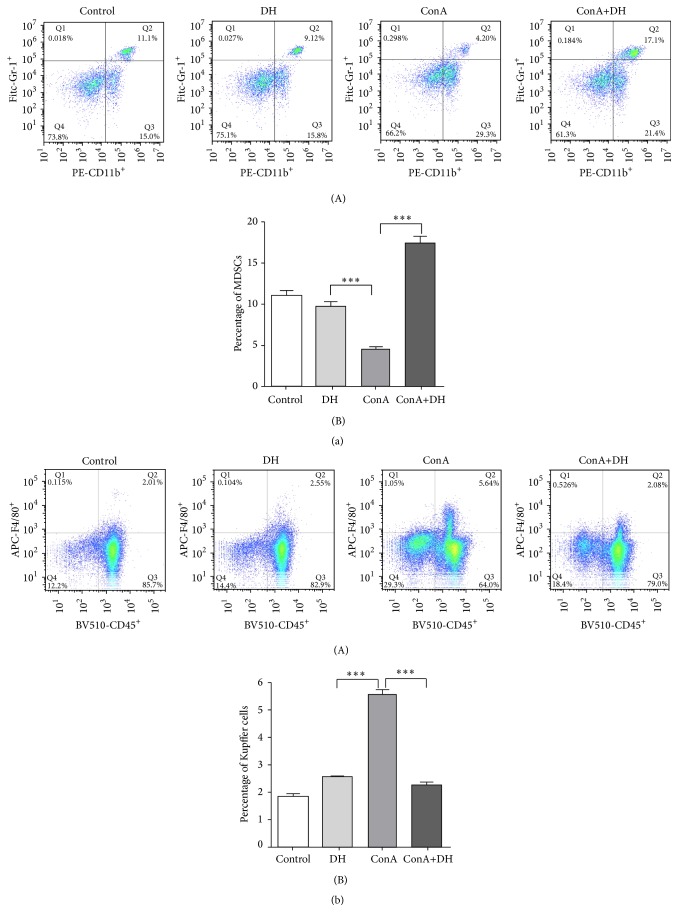
DH recruits more MDSCs to liver and inhibits the infiltration of macrophages in liver. Notes: hepatitis was induced by* i.v.* injection of ConA (15 mg/kg). Liver mononuclear cells were isolated from mice at 18 h following ConA injection. CD11b^+^ Gr1^+^ MDSCs (a) and CD45^+^ F4/80^+^ Kupffer cells (b) were stained and analyzed by flow cytometer. Numbers in quadrants indicate the percentage of cells in each ((a)(A) and (b)(A)). Proportions of CD11b^+^ Gr1^+^ MDSCs (a)(B) and CD45^+^ F4/80^+^ Kupffer cells (b)(B) among total liver mononuclear cell population (mean ± SD *n* = 3; ^*∗∗∗*^ *P* < 0.0001). ConA: Concanavalin A; DH:* Dracocephalum heterophyllum*; MDSCs: myeloid-derived suppressor cells.

MDSCs are a negative regulator of immune cells in maintenance of the immune homeostasis in liver. We explored whether DH affects recruitment and infiltration of MDSCs in liver under inflammatory conditions and found that the percentage of CD11b^+^ Gr-1^+^ MDSCs in DH-pretreated mice liver was significantly higher than that in nonpretreated group (Figure 6(a)). It has also been reported that MDSCs inhibit infiltration of macrophages in the liver. This study seems to agree with those findings, as we found that the percentage of CD45^+^ F4/80^+^ macrophages in ConA group was much higher than that in DH-pretreated group (Figure 6(b)). These results suggest that DH might recruit more MDSCs to liver and inhibit macrophage infiltration and inflammatory responses to reduce liver injury.

## 4. Discussion

Traditional Chinese herbal medicines play an important role in various disease therapies. An increasing number of herbs touted as potential therapeutic protection against liver disease have been reported [15, 16]. DH, one of the touted herbs, has been used in treatment of liver diseases for hundreds of years, but its underlying molecular mechanism has been elusive.

ConA-induced hepatitis is an animal model of immune-meditated liver injury, which shows acute increase of blood transaminases, infiltration of inflammatory cells, and secretion of proinflammatory cytokines [6, 7]. The present study examines the therapeutic effect of DH in ConA-induced acute hepatitis and provides novel evidences regarding its pharmacological properties. Mortality in ConA group injected with lethal dose of ConA at 16 h is 100%, but there is no death in DH-treated mice injected with lethal dose of ConA at 16 h. When ConA injected is reduced to sublethal dose, we found that DH suppressed the ALT/AST levels and decreased liver damage.

Liver damage caused by hepatitis is associated with excessive activation of the immune responses [17]. It has been reported that TNF-*α* and IFN-*γ* participate in various forms of liver damage, such as viral, toxic, and autoimmune hepatitis, and play an important role in ConA-induced hepatitis [18–20]. Our results show that DH can significantly reduce production of IFN-*γ* (Figure 5(a)) and TNF-*α* (Figure 5(b)). It has also been reported that Kupffer cells secrete a large amount of TNF-*α* to aggravate the liver damage [3]. Kupffer cells play an important role in T cell activation-induced liver injury by contributing to IFN-*γ* production. Once Kupffer cells were depleted, liver damage is completely suppressed [3, 21]. In this study, we found that DH effectively suppressed Kupffer cell infiltration in liver (Figure 6(b)).

CD11b^+^ Gr-1^+^ MDSC suppresses the activation and function of Kupffer cells and T cells [22–24]. It has been reported that FTY720, a drug used to treat autoimmune hepatitis, could recruit more MDSC, inhibit Th1 cells to produce IFN-*γ*, activate Foxp3^+^ Tregs, and decrease liver damage in ConA-induced hepatitis [25]. MDSC accumulation enhances the IL-10 secretion and downregulates production of TNF-*α* and IL-12 [26, 27]. Consistent with these results, our results show that DH can recruit more MDSC to the liver and inhibit the activation of macrophages and T cells.

In summary, we used ConA-induced liver injury model to examine the effect of DH on acute hepatitis. The changes in histology, apoptosis, inflammation responses, and immune cell infiltration were observed. The results reveal that DH inhibits inflammatory responses to protect against ConA-induce liver injury.
